# Risk Factors for Prolonged Length of Stay of Older Patients in an Academic Emergency Department: A Retrospective Cohort Study

**DOI:** 10.1155/2019/4937827

**Published:** 2019-05-02

**Authors:** Özcan Sir, Gijs Hesselink, Mara Van Den Bogaert, Reinier P. Akkermans, Yvonne Schoon

**Affiliations:** ^1^Department of Emergency Medicine, Radboud University Medical Center, Nijmegen, Netherlands; ^2^Radboud University Medical Center, Radboud Institute for Health Sciences, IQ Healthcare, Nijmegen, Netherlands; ^3^Department of Geriatrics, Radboud University Medical Center, Nijmegen, Netherlands; ^4^Department of Primary and Community Care, Radboud University Medical Center, Nijmegen, Netherlands; ^5^Radboud University Medical Center, Radboud Institute for Health Sciences, Nijmegen, Netherlands

## Abstract

Emergency departments (EDs) are challenged with a growing population of older patients. These patients are at risk for a prolonged length of stay (LOS) at the ED and face more complications and poorer clinical outcomes. We aimed to identify risk factors for a prolonged LOS of older patients at the ED. For this retrospective clinical database study, we analyzed medical records of 2000 patients ≥70 years old presenting at the ED of a large level I trauma center in the Netherlands. LOS above the 75^th^ percentile of LOS at our ED, 293 minutes, was considered prolonged. After bivariate analysis, we identified associations between LOS and patient, organizational, and clinical factors. Associations with a* p* < 0.05 were inserted in multivariable logistic regression models. We analyzed 1048 men (52%) and 952 women (48%) with a mean age of 78 ± 6.2 years. Risk factors for prolonged LOS of older patients at the ED were follows: higher number (more than one) of consultations (OR [odds ratio] 2.4, CI [confidence interval] 2.0-2.91), or diagnostic interventions (OR 1.5, CI 1.4-1.7); presenting complaints of a neurological (OR 2.2, CI 1.0-4.5) or internal medicine focus (OR 2.6, CI 1.4-4.6); patients with an altered consciousness (OR 3.3, CI 1.6-6.6); treatment by physicians of the departments of surgery (OR 3.4, CI 2.2-5.2), internal medicine (OR 2.6, CI 1.9-3.7), or pulmonology (OR 2.2, CI 1.4-3.6); and urgency category of ≥ U1. Awareness of factors associated with prolonged LOS of older patients presenting at the ED is essential. Physicians should recognize and take these factors into account, in order to improve clinical outcomes of the (strongly increasing) population of older patients at the ED.

## 1. Introduction

The number of older patients attending the emergency department (ED) is increasing due to a growing population with a rising life expectancy [1–3]. Coping with the large number of older patients attending the ED is an international concern for emergency care staff [3]. Older patients often require more tailored care due to an atypical presentation of symptoms, multimorbidity, and concomitant polypharmacy [4, 5]. They also have more complex problems and need more diagnostics and consulting specialists [6]. All these factors potentially contribute to prolonged length of stay (LOS) at the ED. Prolonged LOS of older patients at the ED has been shown to be associated with a higher risk of hospitalization and adverse outcomes [5].

Identifying risk factors for a prolonged ED LOS of older patients may provide insight into possible strategies to decrease LOS of older patients at the ED. Despite the increase of older patients presenting at the ED and thereby contributing to crowding in the ED, relatively few studies have studied risk factors for a prolonged ED LOS of older patients presenting at the ED [7, 8]. Both studies, being well conducted, had an important limitation: the type of medical specialty, as an explanatory factor for prolonged LOS among older patients presenting at the ED, was not included in the analysis. This is in contrast with our experience at a level I trauma center in an urban area in the Netherlands, where we notice marked differences in ED LOS between patients treated by different specialties.

The aim of this study is to identify all patient, organizational, and clinical characteristics that may be associated with LOS of older patients at the ED. Insight into all factors contributing to prolonged LOS of older patients at the ED could provide vital input for developing or choosing strategies to prevent prolonged LOS at the ED, thereby achieving better quality of care for older patients attending the ED.

## 2. Methods and Materials

### 2.1. Study Design, Setting, and Participants

#### 2.1.1. Study Design and Setting

This retrospective cohort study was performed at the ED of the Radboud University Medical Center (Radboudumc), an academic level I trauma center in the Netherlands, with a 650-bed capacity and an annual census of approximately 22,000 patients. The study was carried out in accordance with the regulations governed by the Institutional Review Board of the Radboudumc and exempted from review.

#### 2.1.2. Study Population

We queried our institutional database for all patients who presented at the ED in 2014 (January–December). Patients admitted to the cardiac care unit were excluded, because these patients were all treated by a separate medical team under supervision of a cardiologist.

### 2.2. Data Collection

The values of the variables of interest were digitally extracted out of the hospital's electronic patient record database. Data that required clinical interpretation were collected manually.

#### 2.2.1. Variables

Our variables of interest were the patient characteristics (i.e., age, sex, presence of cognitive impairment, polypharmacy, and Charlson Age-Comorbidity Index), organizational factors (i.e., day of presentation, time of presentation, number of consultations, diagnostic interventions, therapeutic interventions, mode of presentation, method of transport, seniority of physician, assigned urgency, destination after ED visit, and revisit of the ED (after index visit)), and clinical factors (i.e., presenting complaint, diagnosis at ED visit, and treating specialty).

#### 2.2.2. Definitions

ED LOS was defined as time in minutes between arrival and ED discharge or hospital admission. Prolonged ED LOS was ascertained in accordance with the definition by Brouns et al. [7], that is, LOS at the ED larger or equal to the 75^th^ percentile of LOS at the ED. In our total study population prolonged ED LOS was calculated as >293 minutes. Time of presentation was classified as morning (7.00–11.59 h), afternoon (12.00–16.59 h), evening (17.00–23.59 h), and night (0.00–6.59 h). Type of referral was categorized into referral by a general practitioner, practitioner of another hospital, emergency call, physician within the hospital, and self-referral. Method of transport was classified as self-transport, ambulance, medical mobile team (MMT = trauma helicopter), and other methods of transport. Treating specialty at the ED was categorized into six specialties with most patients allocated (emergency physician, surgery, internal medicine, geriatrics, pulmonology, and neurology). All other specialties were classified as “other specialties.” Each type of complaint was categorized into a subgroup of presenting complaints (Table 1). Emergency physicians in our hospital treated all patients, referred to the ED by emergency calls, general practitioner, or self-referral, regardless of the type of presenting complaint. Our ED is 24/7 staffed with emergency physicians. Specialists of other specialties generally attend the ED on request of the emergency physician or the resident. All medical specialties do have a trainee resident or a nontrainee resident available to treat patients primarily or as a consultant. Triage levels were determined by using the Netherlands Triage System (NTS, with U0 being the highest urgency and U5 being the lowest urgency). The Charlson Age-Comorbidity Index was calculated to assess the comorbidity levels (ranged from 0 to 40; higher score means more comorbidity [9]). Polypharmacy was defined as the use of five or more different medications prescribed by a physician. The seniority of the first physician was classified as ED resident, resident of specified other specialism, or emergency physician. Cognitive impairment was assessed by reviewing medical history and ED notes of the day of visit. A preexistent diagnosis of dementia was classified as ‘cognitive impairment.' Notes of emergency physicians and geriatricians were evaluated in terms of ‘unreliable anamnesis,' ‘confusion,' and ‘possible delirium' and, when found, were scored ‘probable cognitive impairment.' In the absence of a preexisting diagnosis of dementia and no notes of suspected cognitive impairment, ‘no cognitive impairment' was noted. The number of (how many) other specialisms that were consulted within one presentation at the ED was counted. Diagnostic interventions consisted of blood examinations, electrocardiogram, ultrasound, X-rays, CT scan, MRI scan, lumbar puncture, puncture of a swollen joint, thoracocentesis, ankle brachial pressure index, and flexible endoscopy by an ENT physician. Radiological imaging with the same type of diagnostics, but on multiple body parts, was counted as one (e.g., X-rays of neck, pelvis, and hip made at one ED visit). Therapeutic interventions comprised (re)placing a urinary catheter, suprapubic catheter, or nasogastric tube, reposition of a fracture or dislocation, sedation (including intubation), thrombolysis, placing a chest tube, or placing a halo frame.

### 2.3. Statistical Analyses

We used frequencies and percentages to describe discrete variables and the mean and standard deviation (SD) to describe continuous variables. We used a dataset consisting of patients with complete medical charts without any missing values of our study variables. We performed bivariate analysis to identify factors to be included in our multivariable analysis. The two-sample t-test was used to identify associations between continuous variables and prolonged ED LOS. The association between discrete variables and ED LOS was assessed by using the Pearson's Chi-square test. We decided to use a* p-*value of < 0.05 in bivariate analysis as an entry criterion for the multivariable model. We checked the correlation of the factors and, when factors were mutually strongly related, a variable was selected for exclusion from the multivariable logistic regression analysis model. Multivariable logistic regression analysis was used to calculate the odds ratio (OR) with 95% confidence intervals (CI) to identify independent risk factors for a prolonged ED LOS.* p*-values < 0.05 were considered statistically significant, based on two-sided tests. The Nagelkerke's R^2^ is calculated for testing the performance of the model.

## 3. Results

### 3.1. Study Population

A total of 22,285 patients visited the ED in 2014. After exclusion of patients younger than 70 years old, 4,781 patients were eligible for the study. We sorted the patients on ascending patient identification number and selected the first 2,000 patients for inclusion in our study. While preparing the database, 92 patients were excluded for a variety of reasons (Table 2). They were substituted by 92 other patients, who were again selected on ascending patient number. Our final study cohort consisted of 2,000 patients.

### 3.2. Demographical, Organizational, and Clinical Characteristics of Patients

The mean age of patients was 78 years (SD 6.2 years). Half of the patients were male (n=1,048; 52%). Most patients had no diagnosis or signs of cognitive impairment (n=1,654; 83%). Polypharmacy existed in two-thirds of the patients (n=1,310; 66%) (Table 3).

About three-quarters of all patients were presented during weekdays (n=1,536; 77%). Almost half of the patients presented in the afternoon (n=980; 49%). The mean number of consultations per patient was 0.47 (SD 0.74) and mean number of diagnostics per patient was 2.2 (SD 1.4). Patients were most frequently admitted by the general practitioner (n=760; 38%). Most patients arrived at the ED by self-transport (n=1,171; 59%). Patients were mainly treated by residents (n=1,678; 84%). About 44% of all patients were triaged as U1-U2 (n= 876). Of the 2,000 patients that visited the ED, almost two-thirds (n=1,251; 63%) were admitted to the hospital. Fourteen patients (0.7%) died at the ED. Fourteen percent of the patients (n=272) revisited the ED within a month after ED discharge.

Most patients had a presenting complaint (n=1,032; 52%) in the field of internal medicine. Final diagnosis was only in 32% of the patients (n=642) in the field of internal medicine. This discrepancy could be explained by the fact that some patients with a presenting complaint in the field of internal medicine had a diagnosis in the field of another specialism (e.g., a patient with upper abdominal pain with a diagnosis of acute coronary syndrome or pneumonia). About one-third of all patients (n=729; 36%) were treated by the emergency physician (Table 3).

### 3.3. Demographical Factors Related to a Prolonged LOS

The median length of stay of our study cohort was 216 minutes (SD 116 minutes). A quarter of the patients (n=505, 25%) had a prolonged length of stay. Polypharmacy was significantly associated with a prolonged ED LOS (*p *< 0.001) in the bivariate analysis (Table 4). However, polypharmacy was not independently related to a prolonged stay at the ED in the multivariable analysis (*p = *0.56; Table 5).

### 3.4. Organizational Factors Independently Related to a Prolonged LOS

Day of presentation (*p *= 0.036), time of presentation (*p *= 0.022), number of consultations (*p *< 0.001), number of diagnostic interventions (*p *< 0.001), mode of presentation (*p *< 0.001), method of transport (*p *= 0.021), seniority of physician (*p *= 0.015), assigned urgency (*p *< 0.001), and destination after ED visit (*p *< 0.001) were significantly univariately associated with ED LOS (Table 4). In multivariable analysis, risk of prolonged ED LOS was higher for patients with a higher number of consultations (OR 2.4, CI 2.0-2.91) or with diagnostic interventions (OR 1.5, CI 1.4-1.7). Patients with an urgency category of U1-U2 (OR 4.8, CI 2.2-10), U3-U4 (OR 6.3, CI 2.9-14), or U5/missing (OR 2.9, CI 1.2-6.8) were more likely to have a prolonged ED LOS than patients with an urgency category of U0 (Table 5).

### 3.5. Clinical Factors Independently Related to a Prolonged Length of Stay at the ED

All clinical factors (i.e., presenting complaint, diagnosis at the ED visit, and the treating specialty) were significantly related to a prolonged ED LOS (all* p *< 0.001; Table 4). In multivariable analysis, presenting complaint of a neurological (OR 2.2, CI 1.0-4.5) or internal medicine focus (OR 2.6, CI 1.4-4.6) had a higher likelihood of a prolonged LOS at the ED when compared to older patients with a presenting complaint of trauma. Older patients, presenting at the ED, with an altered consciousness, also had a higher likelihood of a prolonged LOS at the ED (OR 3.3, CI 1.6-6.6) (Table 5).

When compared to patients treated by an emergency physician, patients treated by physicians of the departments of surgery (OR 3.4, CI 2.2-5.2), internal medicine (OR 2.6, CI 1.9-3.7), or pulmonology (OR 2.2, CI 1.4-3.6) had a significantly prolonged LOS at the ED (Table 5). However, we should take into account that the group of patients treated by the emergency physician contained patients with less comorbidity: 91% of all patients treated by the emergency physicians had a CACI score of 3-7. In contrast, 80% of patients treated by doctors of other specialisms had a CACI score of 3-7. When compared to doctors of other specialisms, emergency physicians less often treated patients with higher CACI scores ranging from 8 to 11 (8.9% vs. 19%) and scores ranging from 12 to 14 (0 vs. 1.4%; P < 0.001).”

## 4. Discussion

In this study we identified several risk factors for a prolonged LOS of older patients at the ED. We found that older patients with presenting complaints of a neurological or internal medicine focus had a higher likelihood of a prolonged LOS at the ED. Patients with an altered consciousness, higher number of consultations, and higher number of diagnostic interventions also had a higher likelihood of prolonged LOS at the ED. Treatment of older patients by physicians of the departments of surgery, internal medicine, or pulmonology were more likely to result in prolonged LOS at the ED.

The association of presenting complaints with prolonged LOS at the ED has been studied in different patient populations [10], but there is little published data on this association specifically in the subgroup of older patients. A recent study that examined this association among older patients [7] did not find an association between complaints at presentation and LOS of older patients at the ED, which is in contrast with our findings. The difference in findings may be explained by the fact that we included complaints related to all medical fields, while they only included complaints at presentation that were related to the field of internal medicine. Different types of presenting complaints may require different clinical approaches, which could justify the difference in LOS at the ED. The association that we found between a higher number of consultations and a prolonged LOS at the ED has been identified before [7, 11], and so has the association between a prolonged LOS at the ED and a higher number of diagnostic interventions [7, 12]. Our study has shown that older patients at the ED often have complex problems, which require more than one treating specialty and several consultations and diagnostic interventions to make a treatment plan at the ED.

Compared to older patients who had an urgency of U0, older patients with a lower urgency category were more likely to have a prolonged LOS. This is in line with the findings of a recent study [8]. We found contrasting findings in prior published data [12, 13], but patients with the population of interest in those studies were not specified on older patients explicitly.

When compared to older patients treated by an emergency physician, older patients who were treated by doctors of the departments of surgery, internal medicine, or pulmonology were more likely to have a prolonged stay at the ED. However, it should be noted that patients with lower comorbidity were treated by emergency physicians more often than by doctors of other specialisms. Patients with lower comorbidity presumably have shorter LOS at the ED. Secondly, in our ED all patients with minor trauma are treated by emergency physicians. These patients usually have presenting complaints, which can be diagnosed and treated rapidly (e.g., a patient with a wound) resulting in a short LOS at the ED. To the best of our knowledge, the association between the type of attending physician and prolonged LOS of older patients at the ED has not been identified before. The finding that the odds of a prolonged LOS may be affected by the type of treating specialty could be a reason for future research on streamlining clinical processes at the ED.

The increase of older patients, who require more extensive and personalized care, challenges EDs to find solutions for the increasing LOS of these patients at the ED. Knowledge of the factors that may affect LOS can help emergency physicians minimize the LOS of older patients at the ED and may reduce the patient complication rate that goes along with a prolonged LOS at the ED [5]. Our results showed that patients with altered consciousness or presenting complaints of neurological or internal medicine focus are prone to prolonged LOS at the ED. Emergency physicians could be trained to enhance their knowledge and competencies in these fields, in an attempt to treat these patients in a better and timely manner and prevent prolonged LOS. Our results also showed that patients treated by doctors of the departments of surgery, internal medicine, or pulmonology were more likely to have a prolonged LOS at the ED. One solution to reduce the LOS of these patients at the ED could be a better collaboration between these specialties and the emergency physicians. Our ED is 24/7 staffed with emergency physicians and could assist the departments of surgery, internal medicine, and pulmonology in order to minimize LOS at the ED. These interventions may also facilitate increasing the capacity to treat more patients with the same resource allocations at ED's and, therefore, improve the availability of quality care for older patients. Prevention of prolonged LOS is not a goal in itself; the group of older patients is vulnerable and susceptible to complications when assessment at the ED is incomplete. Physicians should always be aware of the fact that a prolonged LOS may indicate that a higher level of expert care is needed.

## 5. Limitations

This study should be interpreted in light of its limitations. Since this is a retrospective study, we relied on accurate record keeping by ED workers. The LOS was calculated based on the registered values of time of arrival and time of departure. It could be that, due to prioritizing clinical care over registration logistics, the actual time of arrival and departure may slightly differ from the times registered in the clinical records. Second, we included patients based on ascending patient identification number. Prior to inclusion, we consulted our IT department to evaluate the attribution process of these numbers at our institution. The allotment is not based on chronology and can be considered random, but it is possible that our inclusion process may not have terminated the possibility of selection bias completely based on attribution algorithms of the hospital information system that are unknown to us. Third, a few risk factors for a prolonged LOS at the ED are logical (e.g., multiple consultations and multiple diagnostic interventions). These risk factors could partly be eliminated by performing only the most necessary consultations and diagnostic interventions at the ED. All other consultations and diagnostic interventions could be postponed to the ward. Also, when consultations and diagnostics are indicated to be performed at the ED, this could be done as soon as possible in an attempt to prevent prolonged LOS at the ED. We choose not to exclude these common-sense risk factors for prolonged LOS at the ED, because the intention of our study is to thoroughly examine all potential risk factors for a prolonged LOS at the ED. Fourth, as not all patients are primarily treated by emergency physicians, confounding by indication is an important limitation of this study. Finally, with regard to unplanned revisits to the ED, it could be that patients might have visited another ED and were therefore not registered as a revisit.

## 6. Conclusions

In the current study, we were able to identify several risk factors for prolonged LOS of older patients at the ED. We found that the type of presenting complaints is of influence on the LOS at the ED: older patients with altered consciousness, neurological complaints, or complaints in the field of internal medicine had a higher likelihood of a prolonged LOS at the ED. Patients with a higher number of consultations or higher number of diagnostic interventions also had a higher likelihood of prolonged LOS at the ED. We also found evident differences between medical specialties regarding LOS at the ED: older patients treated by physicians of the departments of surgery, internal medicine, or pulmonology were more likely to have a prolonged LOS at the ED. The results of our study underline that LOS of older patients at the ED depends on organizational and clinical factors. It is necessary that physicians take these factors into consideration in the challenge of avoiding prolonged LOS of older patients at the ED.

## Figures and Tables

**Table 1:** Categories for presenting complaints.

Category	Type of complaints
Traumatic injuries	Pain after trauma, wounds, burns, complaints after falling, osteoporotic vertebral fractures.

Small interventions	Placing a urinary catheter for a newly diagnosed urinary retention, nose bleeds, abscesses.

Neurological complaints	Dizziness, epileptic insult, headache, radiating pain back/leg, neurological paralysis, slurred speech.

Respiratory complaints	Dyspnoea, haemoptysis, cough, suspected pneumonia / pulmonary embolism, pneumosepsis.

Internal medicine	Allergic reaction, anaemia, rash, hypertension, hyper/hypoglycaemia, complaints while on chemotherapy, fever, painful joints without trauma, fatigue, skin infections, pain in eyes or ears, intoxication, septic arthritis.

Abdominal complaints	Vomiting, abdominal ache, diarrhoea, hematemesis, haematuria, icterus, melena, nausea, constipation, rectal blood loss, vaginal blood loss, pain in testes or vulva, suspected kidney stones, pain in groin or side.

Painful or swollen leg	Painful leg or ankle without trauma, suspected deep venous thromboembolism, painful hip without trauma, diabetic foot.

Altered consciousness	Collapse, confused, lowered level of consciousness.

Chest complaints	Chest pain.

Complaints due to medical treatment	All problems following surgery (bleeding, fever, pain, infection), catheter related problems, problems with plaster, casts and bandages, problems with drains and other medical devices.

Resuscitation	In need of resuscitation when arriving at the ED.

ED: emergency department

**Table 2:** Exclusions.

Number of patients	Reason for exclusion	
2	Missing LOS	No LOS could be calculated neither automatically nor manually.

2	Administrative mistake	Patient was registered twice.

21	No or incomplete notes	Missing notes.

26	Observation	Patients kept for observation due to a medical reason or a delay in transfer or logistics.

26	Outpatient department (OPD)	Patients primarily admitted to OPD for ophthalmology, oral and maxillofacial surgery, or ENT. For administrative reasons these patients were registered at the ED; however consultation took place at OPD.

15	Procedure in the ED	Patients coming in for a small procedure not involving a physicians' consultation, e.g., changing or flushing a urinary catheter or cutting a ring.

Total of 92		

ED: emergency department

LOS: length of stay

OPD: outpatient department

ENT: ear nose throat

**Table 3:** Demographical, organizational, and clinical characteristics of patients.

Variable	All patients (n=2000)
*Patient characteristics*	
Age at ED visit, mean ± SD	78 ± 6.2
Sex, n (%)	
Men	1048 (52)
Women	952 (48)
Presence of (signs of) cognitive impairment, n (%)	346 (17)
Polypharmacy, n (%)	1310 (66)
CACI, mean ± SD	5.6 ± 2.1

*Organizational factors*	
Day of presentation, n (%)	
Monday through Friday	1536 (77)
Saturday and Sunday	464 (23)
Time of presentation, n (%)	
Night (00.00 - 6.59)	138 (6.9)
Morning (7.00 - 11.59)	406 (20)
Afternoon (12.00 - 17.59)	980 (49)
Evening (18.00 - 23.59)	476 (24)
Number of consultations per patient, mean ± SD	0.47 ± 0.74
Number of diagnostic interventions per patient, mean ± SD	2.2 ± 1.4
Number of therapeutic interventions per patient, mean ± SD	0.96 ± 0.33
Mode of presentation, n (%)	
General practitioner	760 (38)
Specialist of our institution	561 (28)
Emergency call	447 (22)
Self-referral	157 (7.9)
Another hospital	75 (3.8)
Method of transport, n (%)	
Self-transport	1171 (59)
Ambulance	782 (39)
MMT/trauma helicopter	33 (1.7)
Other	14 (0.70)
Seniority of physician, n (%)	
Resident	1678 (84)
Attending specialist	322 (16)
Assigned urgency, n (%)	
U0	101 (5.1)
U1 - U2	876 (44)
U3 - U4	751 (38)
U5 or missing	272 (14)
Destination after ED visit, n (%)	
Admitted to hospital	1251 (63)
Discharged	735 (37)
Deceased at the ED	14 (0.70)
Revisits, n (%)	
Revisits < 15 days	173 (8.6)
Revisits 15-30 days	99 (5,0)
Revisits < 30 days	272 (13.6)

*Clinical factors*	
Presenting complaint, n (%)	
Internal medicine	1032 (52)
Trauma	329 (16)
Neurological	285 (14)
Altered consciousness	119 (6.0)
Sequelae of medical procedure	112 (5.6)
Other	64 (3.2)
High impact trauma	34 (1.7)
Resuscitation	25 (1.3)
Diagnosis at ED visit, n (%)	
Internal medicine	642 (32)
Neurology	339 (17)
Surgical, other than trauma	325 (16)
Cardiology or pulmonology	308 (15)
Trauma	237 (12)
Other	106 (5.3)
Resuscitation	26 (1.3)
Multitrauma	17 (0.85)
Treating specialty, n (%)	
Emergency physician	729 (36)
Internal medicine	462 (23)
Neurology	216 (11)
Surgery	193 (9.7)
Other	173 (8.7)
Pulmonology	123 (6.2)
Geriatrics	104 (5.2)

CACI: Charlson Age-Comorbidity Index

MMT: medical mobile team

ED: emergency department

**Table 4:** Bivariate analysis: factors associated with a prolonged LOS at the ED (LOS > 293 minutes).

Variable		Prolonged ED-LOS		
	All patients	No	Yes	* p*-value
	(n=2000)	(n=1495)	(n=505)	
*Patient characteristics*				
Age at ED visit, mean ± SD	78 ± 6.2	78 ± 6.4	78 ± 5.9	0.63^1^
Sex, n (%)				0.79^2^
Men	1048 (52)	786 (53)	262 (52)	
Women	952 (48)	709 (47)	243 (48)	
Presence of cognitive impairment, n (%)	346 (17)	256 (17)	90 (18)	0.72^2^
Polypharmacy, n (%)	1310 (66)	945 (63)	365 (72)	**<** *0.001* ^*2*^
CACI, mean ± SD	5.6 ± 2.1	5.5 ± 2.1	5.6 ± 2.1	0.46^1^

*Organizational and logistical factors*				
Day of presentation, n (%)				*0.036* ^*2*^
Monday through Friday	1536 (77)	1131 (76)	405 (80)	
Saturday and Sunday	464 (23)	364 (24)	100 (20)	
Time of presentation, n (%)				*0.022* ^*2*^
Night (00.00 - 6.59)	138 (6.9)	108 (7.2)	30 (5.9)	
Morning (7.00 - 11.59)	406 (20)	306 (20)	100 (20)	
Afternoon (12.00 - 17.59)	980 (49)	705 (47)	275 (54)	
Evening (18.00 - 23.59)	476 (24)	376 (25)	100 (20)	
Number of consultations per patient, mean ± SD	0.47 ± 0.74	0.34 ± 0.63	0.84 ± 0.90	*<0.001* ^*1*^
Number of diagnostic interventions per patient, mean ± SD	2.2 ± 1.4	2.0 ± 1.3	2.9 ± 1.3	*<0.001* ^*1*^
Number of therapeutic interventions per patient, mean ± SD	0.096 ± 0.33	0.10 ± 0.34	0.07 ± 0.29	0.071^1^
Mode of presentation, n (%)				*<0.001* ^*2*^
General practitioner	760 (38)	538 (36)	222 (44)	
Specialist of our institution	561 (28)	425 (28)	136 (27)	
Emergency call	447 (22)	333 (22)	114 (23)	
Self-referral	157 (7.9)	134 (9.0)	23 (4.6)	
Another hospital	75 (3.8)	65 (4.4)	10 (2.0)	
Method of transport, n (%)				*0.021* ^*2*^
Self-transport	1171 (59)	862 (58)	309 (61)	
Ambulance	782 (39)	590 (39)	192 (38)	
MMT/trauma helicopter	33 (1.7)	32 (2.1)	1 (0.20)	
Other	14 (0.70)	11 (0.74)	3 (0.59)	
Seniority of physician, n (%)				*0.015* ^*2*^
Resident	1678 (84)	1237 (83)	441 (87)	
Attending specialist	322 (16)	258 (17)	64 (13)	
Assigned urgency, n (%)				*<0.001* ^*2*^
U0	101 (5.1)	91 (6.1)	10 (2.0)	
U1 - U2	876 (44)	625 (42)	251 (50)	
U3 - U4	751 (38)	537 (36)	214 (42)	
U5 or missing	272 (14)	242 (16)	30 (5.9)	
Destination after ED visit, n (%)				*<0.001* ^*2*^
Admitted to hospital	1251 (63)	860 (58)	391 (77)	
Discharged	735 (37)	624 (42)	111 (22)	
Deceased at the ED	14 (0.70)	11 (0.74)	3 (0.59)	

*Clinical factors*				
Presenting complaint, n (%)				*<0.001* ^*2*^
Internal medicine	1032 (52)	720 (48)	312 (62)	
Trauma	329 (16)	270 (18)	59 (12)	
Neurological	285 (14)	228 (15)	57 (11)	
Altered consciousness	119 (6.0)	79 (5.3)	40 (7.9)	
Sequelae of medical procedure	112 (5.6)	98 (6.6)	14 (2.8)	
Other	64 (3.2)	49 (3.3)	15 (3.0)	
High impact trauma	34 (1.7)	29 (1.9)	5 (0.99)	
Resuscitation	25 (1.3)	22 (1.5)	3 (0.59)	
Diagnosis at ED visit, n (%)				*<0.001* ^*2*^
Internal medicine	642 (32)	441 (30)	201 (40)	
Neurology	339 (17)	275 (18)	64 (13)	
Surgical, other than trauma	325 (16)	237 (16)	88 (17)	
Cardiology or pulmonology	308 (15)	207 (14)	101 (20)	
Trauma	237 (12)	197 (13)	40 (7.9)	
Other	106 (5.3)	99 (6.6)	7 (1.4)	
Resuscitation	26 (1.3)	23 (1.5)	3 (0.59)	
Multitrauma	17 (0.85)	16 (1.1)	1 (0.2)	
Treating specialty, n (%)				*<0.001* ^*2*^
Emergency physician	729 (36)	569 (38)	160 (32)	
Internal medicine	462 (23)	295 (20)	167 (33)	
Neurology	216 (11)	173 (12)	43 (8.5)	
Surgery	193 (9.7)	134 (9.0)	59 (12)	
Other	173 (8.7)	153 (10)	20 (4.0)	
Pulmonology	123 (6.2)	77 (5.2)	46 (9.1)	
Geriatrics	104 (5.2)	94 (6.3)	10 (2.0)	

^1^Student t-test; ^2^Chi square

LOS: length of stay

ED: emergency department

CACI: Charlson Age-Comorbidity Index

**Table 5:** Multivariable analysis: independent risk factors for a prolonged LOS (LOS > 293 minutes) at the ED^*∗*^.

			95% CI		
	Odds Ratio	Standard Error	Lower	Upper	* p-*value
*Patient characteristics*					
Age, per year increase	1.0	0.0096	0.98	1.0	0.66
Female sex (ref = male)	1.1	0.13	0.85	1.4	0.56
Polypharmacy	1.1	0.14	0.84	1.4	0.56

*Organizational factors*					
Presentation on weekend (ref = midweek)	0.75	0.11	0.57	1.0	0.051
Time of presentation (ref = morning)					
Night (00.00 - 6.59)	0.66	0.18	0.39	1.1	0.13
Afternoon (12.00 - 17.59)	1.2	0.19	0.89	1.6	0.23
Evening (18.00 - 23.59)	0.85	0.15	0.59	1.2	0.37
Number of consultations, per 1 consultation increase	2.4	0.22	2.0	2.9	*<0.001*
Number of diagnostic interventions, per additional intervention	1.5	0.080	1.4	1.7	*<0.001*
Assigned urgency (ref = U0)					
U1 - U2	4.8	1.9	2.2	10	*<0.001*
U3 - U4	6.3	2.5	2.9	14	*<0.001*
U5 or missing	2.9	1.3	1.2	6.8	*0.016*

*Clinical factors*					
Presenting complaint (ref = trauma)					
High energetic trauma	3.1	2.4	0.71	14	0.13
Resuscitation	9.8	18	0.27	356	0.21
Neurological	2.2	0.81	1.0	4.5	*0.038*
Internal medicine	2.6	0.77	1.4	4.6	*0.002*
Altered consciousness: collapse or confused	3.3	1.2	1.6	6.6	*0.001*
Sequelae of medical procedure	1.7	0.72	0.71	3.9	0.25
Other than the above	3.3	1.6	1.3	8.4	*0.015*
Treating specialty (ref = emergency physician)					
Surgery	3.4	0.75	2.2	5.2	*<0.001*
Internal medicine	2.6	0.46	1.9	3.7	*<0.001*
Geriatrics	0.66	0.25	0.32	1.4	0.26
Pulmonology	2.2	0.54	1.4	3.6	*<0.001*
Neurology	1.5	0.34	0.98	2.3	0.064
Other than the above	1.0	0.30	0.57	1.8	0.96

*∗*Area under the receiver operating characteristic curve: 0.79; pseudo R2, 0.18.

LOS: length of stay

ED: emergency department

## Data Availability

The data used to support the findings of this study are available from the corresponding author upon request.

## References

[1] Aminzadeh F., Dalziel W. B. (2002). Older adults in the emergency department: a systematic review of patterns of use, adverse outcomes, and effectiveness of interventions. *Annals of Emergency Medicine*.

[2] Roberts D. C., McKay M. P., Shaffer A. (2008). Increasing rates of emergency department visits for elderly patients in the United States, 1993 to 2003. *Annals of Emergency Medicine*.

[3] Di Somma S., Paladino L., Vaughan L., Lalle I., Magrini L., Magnanti M. (2015). Overcrowding in emergency department: an international issue. *Internal and Emergency Medicine*.

[4] Limpawattana P., Phungoen P., Mitsungnern T., Laosuangkoon W., Tansangworn N. (2016). Atypical presentations of older adults at the emergency department and associated factors. *Archives of Gerontology and Geriatrics*.

[5] Ackroyd-Stolarz S., Read Guernsey J., MacKinnon N. J., Kovacs G. (2011). The association between a prolonged stay in the emergency department and adverse events in older patients admitted to hospital: a retrospective cohort study. *BMJ Quality & Safety*.

[6] Samaras N., Chevalley T., Samaras D., Gold G. (2010). Older patients in the emergency department: a review. *Annals of Emergency Medicine*.

[7] Brouns S. H. A., Stassen P. M., Lambooij S. L. E., Dieleman J., Vanderfeesten I. T. P., Haak H. R. (2015). Organisational factors induce prolonged emergency department length of stay in elderly patients--a retrospective cohort study. *PLoS ONE*.

[8] Street M., Mohebbi M., Berry D., Cross A., Considine J. (2018). Influences on emergency department length of stay for older people. *European Journal of Emergency Medicine*.

[9] Charlson M., Szatrowski T. P., Peterson J., Gold J. (1994). Validation of a combined comorbidity index. *Journal of Clinical Epidemiology*.

[10] Vegting I. L., Nanayakkara P. W. B., van Dongen A. E. (2011). Analysing completion times in an academic emergency department: coordination of care is the weakest link. *The Netherlands Journal of Medicine*.

[11] Herring A., Wilper A., Himmelstein D. U. (2009). Increasing length of stay among adult visits to U.S. Emergency departments, 2001–2005. *Academic Emergency Medicine*.

[12] McCarthy M. L., Ding R., Pines J. M., Zeger S. L. (2011). Comparison of methods for measuring crowding and its effects on length of stay in the emergency department. *Academic Emergency Medicine*.

[13] Lowthian J., Straney L. D., Brand C. A. (2016). Unplanned early return to the emergency department by older patients: the safe elderly emergency department discharge (SEED) project. *Age and Ageing*.

